# Response to Fluvoxamine in the Obsessive-Compulsive Disorder Patients: Bayesian Ordinal Quantile Regression

**DOI:** 10.2174/1745017902117010151

**Published:** 2021-10-15

**Authors:** Samad Safiloo, Yadollah Mehrabi, Sareh Asadi, Soheila Khodakarim

**Affiliations:** 1 School of Allied Medical Sciences, Shahid Beheshti University of Medical Sciences, Tehran, Iran; 2 School of Public Health and Safety, Shahid Beheshti University of Medical Sciences, Tehran, Iran; 3 Neurobiology Research Center, Shahid Beheshti University of Medical Sciences, Tehran, Iran; 4 School of Medicine, Shiraz University of Medical Sciences, Shiraz, Iran

**Keywords:** Obsessive-compulsive disorder, Fluvoxamine, Ordinal variables, Quantile regression, Chronic neuropsychiatric disorder, SSRIs

## Abstract

**Background:**

Obsessive-Compulsive Disorder (OCD) is a chronic neuropsychiatric disorder associated with unpleasant thoughts or mental images, making the patient repeat physical or mental behaviors to relieve discomfort. 40-60% of patients do not respond to Serotonin Reuptake Inhibitors, including fluvoxamine therapy.

**Introduction:**

The aim of the study is to identify the predictors of fluvoxamine therapy in OCD patients by Bayesian Ordinal Quantile Regression Model.

**Methods:**

This study was performed on 109 patients with OCD. Three methods, including Bayesian ordinal quantile, probit, and logistic regression models, were applied to identify predictors of response to fluvoxamine. The accuracy and weighted kappa were used to evaluate these models.

**Results:**

Our result showed that rs3780413 (mean=-0.69, sd=0.39) and cleaning dimension (mean=-0.61, sd=0.20) had reverse effects on response to fluvoxamine therapy in Bayesian ordinal probit and logistic regression models. In the 75^th^ quantile regression model, marital status (mean=1.62, sd=0.47) and family history (mean=1.33, sd=0.61) had a direct effect, and cleaning (mean=-1.10, sd=0.37) and somatic (mean=-0.58, sd=0.27) dimensions had reverse effects on response to fluvoxamine therapy.

**Conclusion:**

Response to fluvoxamine is a multifactorial problem and can be different in the levels of socio-demographic, genetic, and clinical predictors. Marital status, familial history, cleaning, and somatic dimensions are associated with response to fluvoxamine therapy.

## 1 INTRODUCTION

Obsessive-compulsive disorder (OCD) is a chronic neuropsychiatric disorder associated with unpleasant thoughts or mental images that force the patient to repeat physical or mental behaviors to relieve discomfort [1]. The prevalence of OCD globally is estimated to be 1.5-3%, independent of ethnicity and cultural groups [2]. It is predicted that OCD could be one of the top ten disorders that lead to disability in the next twenty years [3]. The first-line medication to treat this disorder is a class of selective serotonin reuptake inhibitors (SSRIs) which 40-60% of patients do not usually respond to. Therefore, the researchers have focused on the relationship between genetic, clinical, and environmental factors responding to SSRIs [4-11].

The results of previous studies showed inconsistencies in the relationship between the demographic, clinical, and genetic factors with response to SSRIs therapy in OCD patients [12-17]. Storch and *et al*. found an association between the response to fluoxetine in a longer period of illness, older age, and more severe symptoms in OCD patients [15]. On the other hand, Denys *et al*. revealed that patients with no previous history of treatment, moderate severity of OCD, and a low score on the Hamilton Depression Scale were more likely to respond positively to treatment [9]. Hollander *et al*. showed that left-sided visuospatial soft signs significantly increased in the non-responders compared to the responders [18]. Nakatani *et al*. indicated that patients with lower baseline Yale-Brown Obsessive Compulsive Scale (Y-BOCS) and without the cleansing compulsion responded better to treatment [11]. A meta-analysis confirmed the relationship between hoarding, a dimension of OCD symptoms, and poor response to treatment [19].

Although Bella *et al*. could not show the relationship between 5-HTTLPR genotypes and response to fluvoxamine [20], response to SRIs therapy in OCD patients has been revealed to be associated with 5-HTTLPR, 5-HTT, 5HT2A, 5HT1B, BDNF genes in other studies [7, 13, 21].

In the present study, we aimed to investigate the association between the demographic, clinical, and genetic factors with Fluvoxamine therapy in Iranian OCD patients. Here, response to treatment was considered an ordinal variable by three categories: refractory, non-responder, and responder. The ordinal probit or logistic models have usually been proposed for modeling the ordinal outcomes. Both models focus on the cumulative probabilities of the ordinal response variable to take into account the ordinality between the categories in the modeling [22]. We know the validity of these models depends on the assumptions made on the underlying distribution of the latent variable. The two mentioned models do not provide sufficient information about the underlying distribution of the latent variable [23].

In addition to these two models, we used Bayesian quantile ordinal regression model to find predictors of fluvoxamine therapy in Iranian OCD patients.

## 2 MATERIALS AND METHODS

### 2.1 Study Population

In the present study, we used information of 109 OCD patients referred to Imam Hossein Hospital in Tehran, Iran, between 2014 and 2017. They gave consent for their information to be used in the research. Ethical approval for this study was obtained from ethics committee of the Public Health School and Neurosciences Research Center in Shahid Beheshti University of Medical Sciences (ethical code: IR.SBMU.PHNS.REC.1399.013.). More information about the dataset is available in the article of Hasanpour *et al*. [24]. Briefly, the diagnosis of OCD was made according to the fourth edition of the Diagnostic and Statistical Manual of Mental Disorders (DSM-IV-TR) criteria by an expert psychiatrist. Patients with other comorbidities except for depression, anxiety, or tic disorder were excluded from the study. Patients received fluvoxamine during their treatment period (150mg-300mg). The severity of obsessive and compulsive symptoms was evaluated before and after 12 weeks’ treatment with fluvoxamine using the Y-BOCS severity scale. Patients with more than 35% reduction in Y-BOCS scores after treatment were considered as responders, and those with less than 35% reduction in Y-BOCS score were assigned as non-responders. The third group was refractory patients who experienced various selective serotonin reuptake inhibitor (SSRI) trials at the maximum tolerated dose during their illness period but did not respond to them adequately. Hence, response to pharmacotherapy has three ordered categories: 1. Refractory 2. Non-responder 3. Responder.

### 2.2 Data Variables

The socio-demographic variables were age, gender, educational status, occupation, marital status, ethnicity, and the clinical variables included family history of OCD or other mental illnesses, age of disorder onset (under or over 18 years old), and duration of illness.

Moreover, we used five dimensions, including aggression /checking,contamination/cleaning,symmetry/ordering/repeating/ counting/hoarding, sexual and somatic, which have been explored from the Y-BOCS checklist by Asadi *et al*. [25]. In each dimension, the positive and greater values indicate greater severity of the obsession and compulsion in an OCD patient.

### 2.3 Experimental

The salting out method was used to extract genomic DNA from peripheral leucocytes. PCR-RFLP method was used to determine genetic variants of the studied variations. Genotypes for each polymorphism were also confirmed by Sanger sequencing of PCR products [24]. The genetic variables included 5-HTTLPR polymorphism of *SLC6A4* gene, *HTR2A* gene SNPs (rs6311, rs6313), and *SLC1A1* gene SNPs (rs301430, rs3780413, and rs2228622).

### 2.4 Statistical Analysis

Statistical analyses were performed using R software version 3.6.3 (R Foundation for Statistical Computing, Vienna, Austria) and Matlab 2016b. The continuous variables were expressed as mean and standard deviation, and the categorical variables were expressed as frequency and percentages. In order to identify the predictors of pharmacotherapy in OCD patients (ordinal response variable), we used Bayesian ordinal quantile regression [26], Bayesian ordinal probit regression [27], and Bayesian ordinal logistic regression [28] models (Tables **S1** and **S2**). The Bayesian approach could cover the lack of information due to the small sample size by using prior information on the regression coefficients. In all of the mentioned models, the normal prior distribution with large variance (non-informative prior distribution) is considered for estimating the regression coefficients. The inference was made based on 12000 iterations after 3000 were burned. The performance of all the models was evaluated using the Akaike Information Criteria (AIC), weighted kappa, and accuracy.

## 3 RESULTS

Table **1** indicates the distribution of the socio-demographic, clinical variables, and polymorphisms of the selected genes by three categories of the pharmacotherapy results in OCD patients. 64% (70) of patients in the study were female, 72% (79) of patients reported undergraduate education, 57% (62) reported unemployed patients, 83% (91) reported family history of OCD or other mental diseases, 64% (68) of patients had more than 5 years of disease duration, and 66% (72) of patients had been diagnosed after the age of 18. Table **2** indicates the posterior means and standard deviations of parameters in three simple Bayesian ordinal regression models: logistic, probit, and quantile for P 0.25, 0.50 and 0.75.

In order to find the final model (Table **3**) in each approach, all the covariates by a p-value less than 0.2 in Table **2** were included in the model at the beginning and were removed one by one according to a significance criterion (p-value< 0.10). The performance indexes AIC, weighted kappa, and accuracy of each model are reported in Table **3**.

In Bayesian ordinal quantile regression models, contamination/cleaning dimension has a negative effect on the probability of response to fluvoxamine treatment in the OCD patients, especially in the upper half of the distribution. Therefore individuals with compulsive washing responded less to this treatment. The Bayesian ordinal probit and logistic regression models confirmed this subject.

In the upper quartile of the distribution, somatic obsessions had a negative effect on the probability of response to fluvoxamine treatment in OCD patients, which means this treatment was not beneficial for patients with somatic obsessions.

Although Bayesian ordinal probit and logistic regression models could not show any relationship between marital status and family history with response to fluvoxamine treatment in the OCD patients, in the upper half of the distribution, marriage and in the upper quartile of the distribution, family history had a positive effect on this treatment.

Results show that rs3780413 has a negative effect on the response to fluvoxamine treatment in OCD patients in bayesian ordinal probit and logistic regression models.

Choosing the best model in this dataset was not easy. Although the Bayesian ordinal probit and logistic regression models (included rs3780413, Contamination/ Cleaning) have the biggest values of the accuracy and weighted kappa and were the simplest models, they could not find any relationship between socio-demographic variables and the response to fluvoxamine treatment in the OCD patients.

## 4 DISCUSSION

The current study takes into account the simultaneous effects of environmental, genetic, and clinical factors on fluvoxamine therapy and uses three ordinal regression models to identify fluvoxamine therapy predictors. Although we could not see the significant difference among the performance characteristics of these models including accuracy and weighted kappa, the predictors staying in each model were not similar. The socio-demographic predictors played important roles in quantile regression models that could be a proxy of the inequality distribution of these predictors in the population. The ordinal probit and logistic identified rs3780413 had a significant effect on fluvoxamine therapy in OCD patients. Our results could not confirm the findings of Rahman 2016 that shows the Bayesian quantile ordinal regression models provide a better model fit relative to the ordinal probit model [26]. We observed that the model included marital status, familial history, contamination/cleaning, and somatic dimensions. The 75^th^ quantile regression model had a better performance than ordinal logistic and probit regression models, and in the model which included rs3780413 and contamination/cleaning dimensions, ordinal logistic and probit regression models had performed better than 75^th^ quantile regression model (Tables **S1** and **S2**).

Nakatani *et al*. and Mataix-Cols *et al*. showed that the patients without contamination/cleaning and hording dimension had a better response to treatment with fluvoxamine/SSRIs in separate studies [11, 29]. Living with a partner and age of symptom onset were not associated with better response to clomipramine pharmacotherapy in Shavitt *et al*. [14]. Having a family history of anxiety disorders has been shown to have a positive effect on response to the drug [10]. Based on these studies, it will be difficult to choose a model with two predictors as the final model.

Based on previous studies [30-34], several genetic markers were found to be significantly associated with an antidepressant response: *CYP2D6*, *CYP2C19*, *SLC6A4* (5HTTLPR and STin2), *HTR1A* (rs6295), *HTR2A* (rs7997012, rs6311, rs6313, and rs6314), *SLC6A2* (rs5569), *TPH1* (rs1800532). In this study, we investigated the effect of 5-HTTLPR polymorphisms of *SLC6A4* gene, *HTR2A* gene SNPs (rs6311, rs6313), and *SLC1A1* gene SNPs (rs301430, rs3780413, and rs2228622). Our study revealed that *SLC1A1* polymorphism, rs3780413, is associated with response to fluvoxamine therapy.

## Figures and Tables

**Table 1:** Demographic and genetic factors by classification of response to fluvoxamine pharmacotherapy.

**Variables**	**Level**	**Total**	**Sub groups **		
			**Refractory**	**Non-Responder**	**Responder**
		No.	No.	No.	No.
Sex	Male	39.00	7.00	10.00	22.00
	Female	70.00	12.00	18.00	40.00
Marital status	Married	75.00	13.00	16.00	46.00
	Single	34.00	6.00	12.00	16.00
Occupation	Unemployed	62.00	13.00	15.00	34.00
	Employed	47.00	6.00	13.00	28.00
Age of onset	Early	37.00	9.00	8.00	20.00
	Late	72.00	10.00	20.00	42.00
Familial history	Yes	91.00	16.00	21.00	54.00
	no	18.00	3.00	7.00	8.00
Ethnicity	Persian	62.00	11.00	16.00	35.00
	Other	47.00	8.00	12.00	27.00
Education	Academic	30.00	2.00	10.00	18.00
	Under academic	79.00	17.00	18.00	44.00
Illness duration	Under 5 years	41.00	4.00	12.00	25.00
	Upper 5 years	68.00	15.00	16.00	37.00
5-HTTLPR	LL, LS	69.00	12.00	18.00	39.00
	SS	40.00	7.00	10.00	23.00
rs6311	CC, CT	77.00	15.00	15.00	47.00
	TT	32.00	4.00	13.00	15.00
rs6313	CC, CT	81.00	15.00	22.00	44.00
	TT	28.00	4.00	6.00	18.00
rs301430	CC, TC	51.00	9.00	16.00	26.00
	TT	58.00	10.00	12.00	36.00
rs3780413	CC, CG	56.00	3.00	19.00	34.00
	GG	53.00	16.00	9.00	28.00
rs2228622	GA, AA	77.00	14.00	18.00	45.00
	GG	32.00	5.00	10.00	17.00
		Mean(SD)	Mean(SD)	Mean(SD)	Mean(SD)
Age		34.10(9.80)	37.51(2.60)	31.30(7.60)	34.30(9.50)
Aggression/Checking		0.05	-0.20	0.32	0.01
		(1.04)	(1.33)	(1.12)	(0.88)
Contamination/Cleaning		-0.05	0.45	0.12	-0.28
		(96)	(1.06)	(1.08)	(0.80)
Symmetry/Ordering/Counting/Repeating/Hoarding		-0.06	-0.38	0.09	-0.03
		(1.01)	(0.63)	(1.19)	(1.02)
Sexual		-0.04	-0.16	0.19	-0.11
		(0.98)	(1.25)	(0.83)	(0.95)
Somatic		0.01	0.34	0.13	-0.15
		(1.07)	(1.10)	(1.17)	(1.00)

SD: Standard Deviation

**Table 2:** The Results of simple bayesian quantile, probit and logistic models.

** **	**Quantile**									**Ordinal Probit**			**Ordinal Logistic**		
**Variables**	**25^th^**			**50^th^**			**75^th^ e**								
	**Mean**	**SD**	**P**	**Mean**	**SD**	**P**	**Mean**	**SD**	**P**	**Mean**	**SD**	**P**	**Mean**	**SD**	**P**
Sex (male)	0.08	0.7	0.91	0.38	0.62	0.54	0.45	0.55	0.41	-0.02	0.23	0.92	-0.04	0.39	0.93
Marital Status (married)	0.43	0.5	0.39	1.39	0.46	<0.01	1.79	0.5	<0.01	0.24	0.24	0.32	0.43	0.39	0.27
Occupation (unemployed)	0.04	0.5	0.93	0.9	0.5	0.7	1.29	0.55	0.02	-0.19	0.23	0.41	-0.28	0.38	0.46
Age of onset (late)	0.55	0.49	0.26	1.19	0.48	0.01	1.52	0.51	<0.01	0.21	0.24	0.38	0.31	0.4	0.24
Familial history (positive)	0.4	0.67	0.55	1.4	0.63	0.03	1.46	0.57	0.01	0.23	0.29	0.44	0.41	0.47	0.38
Ethnicity (other)	0.32	0.53	0.55	1.09	0.52	0.03	1.48	0.55	<0.01	0.03	0.23	0.9	0.04	0.38	0.9
Education (Academic)	0.7	0.54	0.2	1.25	0.57	0.03	1.72	0.62	<0.01	0.27	0.25	0.29	0.37	0.42	0.38
Illness duration (>5)	-0.06	0.49	0.9	0.86	0.48	0.07	1.25	0.52	0.02	-0.27	0.19	0.24	0.4	0.39	0.3
5-HTTLPR (SS)	0.14	0.7	0.84	0.45	0.62	0.47	0.53	0.55	0.34	0.03	0.23	0.95	0.03	0.39	0.94
rs6311 (TT)	0.17	0.68	0.8	-0.24	0.61	0.7	-0.17	0.52	0.72	-0.14	0.24	0.56	-0.31	0.4	0.43
rs6313 (TT)	0.47	0.75	0.53	0.79	0.67	0.24	0.87	0.6	0.15	0.23	0.26	0.38	0.39	0.44	0.37
rs301430 (TT)	0.38	68	0.58	1.04	0.58	0.07	1.06	0.54	0.05	0.19	0.22	0.39	0.35	0.37	0.34
rs3780413 (GG)	-1.1	0.64	<0.01	0.08	0.6	0.89	0.23	0.53	0.66	-0.46	0.23	0.04	-0.66	0.38	0.08
rs2228622 (GG)	0.13	0.7	0.86	0.14	0.64	0.83	0.21	0.57	0.71	-0.06	0.25	0.83	-0.12	0.4	0.76
Age	0.003	0.03	0.92	0.08	0.03	<0.01	0.12	0.04	<0.01	-0.006	0.01	0.59	-0.007	0.02	0.72
Aggression/Checking	0.2	0.39	0.61	-0.14	0.37	0.7	-0.12	0.31	0.69	0.01	0.1	0.92	-0.01	0.18	0.95
Contamination/Cleaning	-1.05	0.45	0.02	-1	0.36	<0.01	-0.83	0.3	<0.01	-0.36	0.12	<0.01	-0.6	0.2	<0.01
Symmetry/Ordering/…	0.42	0.41	0.31	0.11	0.45	0.81	0.09	0.28	0.75	0.1	0.11	0.37	0.13	0.18	0.46
Sexual	0.06	0.48	0.9	-0.35	0.41	0.39	-0.16	0.27	0.39	-0.04	0.11	0.72	-0.1	0.19	0.61
Somatic	-0.64	0.44	0.15	-0.58	0.35	0.09	-0.35	0.25	0.16	-0.19	0.1	0.06	-0.32	0.17	0.07

SD: Standard Deviation, P: P-value

**Table 3:** The results of multiple bayesian quantile, probit and logistic models.

** **	**Quantile**									**Ordinal Probit**			**Ordinal Logistic**		
**Variables**	**25^th^**			**50^th^**			**75^th^**								
	**Mean**	**SD**	**P**	**Mean**	**SD**	**P**	**Mean**	**SD**	**P**	**Mean**	**SD**	**P**	**Mean**	**SD**	**P**
Marital status (married)	-	-	-	1.12	0.49	0.02	1.62	0.47	<0.01	-	-	-	-	-	**-**
Familial history (positive)	-	-	-	1.08	0.66	0.1	1.33	0.61	0.02	-	-	-	-	-	**-**
rs3780413 (GG)	-1.03	0.65	0.11	-	-	-	-	-	-	-0.45	0.23	0.04	-0.69	0.39	0.07
Contamination/ Cleaning	-0.94	0.42	0.03	-1.14	0.38	<0.01	-1.1	0.37	<0.01	-0.36	0.12	<0.01	-0.61	0.2	<0.01
Somatic	-	-	-	-	-		-0.58	0.28	0.04	-	-	-	-	-	**-**
**GOF index**	Value			Value			Value			Value			Value		
AIC	206.95			207.17			204.18			210.59			211.37		
Weighted kappa	0.13			0.1			0.23			0.3			0.29		
Accuracy	0.587			0.587			0.614			0.614			0.614		

## Data Availability

The data that support the findings of this study are available from the corresponding author, [S.K], upon reasonable request.
